# Resolvin E1's Antimicrobial Potential Against *Aggregatibacter Actinomycetemcomitans*

**DOI:** 10.3389/froh.2022.875047

**Published:** 2022-04-27

**Authors:** Fahad A. Abdullatif, Basmah Almaarik, Mansour Al-Askar

**Affiliations:** ^1^Department of Periodontics and Community Dentistry, College of Dentistry, King Saud University, Riyadh, Saudi Arabia; ^2^Clinical Laboratory Sciences Department, College of Applied Medical Sciences, King Saud University, Riyadh, Saudi Arabia

**Keywords:** Resolvin E1 (RvE1), peri-implantitis treatment, antimicobacterial, *A. actinomycetemcomitans*, oral health related quality of life (OHQoL)

## Abstract

**Background:**

Microorganisms along with host response play a key role in the development of periodontal and peri-implant infections. Advanced periodontal and peri-implant diseases are most likely associated with bacterial plaques that trigger host immune response and eventually lead to the destruction of the attachment apparatus and bone loss around a tooth or a dental implant. A recent systematic review and meta-analysis revealed that *Aggregatibacter actinomycetemcomitans* had the highest association with peri-implantitis. Resolvin E1 (RvE1) is part of the specialized pro-resolving lipid mediator family biosynthesized from omega-3, polyunsaturated fatty acids (PUFAs), and eicosapentaenoic acid (EPA). Although RvE1 is an established anti-inflammatory agent, it was found that its application as a treatment or as a preventive drug had an indirect effect on the subgingival microbiota of both rats and rabbits with experimental periodontitis.

**Aim:**

The aim of this study is to evaluate the direct antimicrobial effect of RvE1 on *Aggregatibacter actinomycetemcomitans* bacteria.

**Materials and Methods:**

The study comprised three groups that underwent minimum inhibitory concentration (MIC) against *Aggregatibacter actinomycetemcomitans*. The first group was tested with the RvE1 working concentration of 5 ug/ml, the second group was tested with ethanol (EtOH), 10% as the working concentration, and the final group was diluted in phosphate-buffered saline (PBS) as the positive control. Optical density (OD_600_) was used for the comparison of bacterial growth among the tested groups. The experiment was conducted in three biological replicates. Data were analyzed using SPSS, and results were analyzed by using one-way analysis of variance (ANOVA) followed by *post-hoc* Bonferroni using a minimum level of significance (*P*-value) of 0.05.

**Results:**

Minimum inhibitory concentration was 1.25 μg/ml and 5% for RvE1 and EtOH, respectively. RvE1's mean optical density (OD_600_) was 0.156 ± 0.021 and was significantly lower compared with all the other groups (*P*-value < 0.01). The EtOH group (mean OD_600_ 0.178 ± 0.013) and the PBS group (mean OD_600_ 0.1855 ± 0.022) did not reveal a significant difference (*P*-value = 0.185).

**Conclusion:**

RvE1 demonstrated significant antimicrobial activity against *A. actinomycetemcomitans* with an MIC of 1.25 μg/ml. The RvE1 group showed significantly lower bacterial growth compared to the EtOH and PBS groups.

## Introduction

Microorganisms along with host response play a key role in the development of periodontal and peri-implant infections. Advanced periodontal and peri-implant diseases are most likely associated with bacterial plaques that trigger host immune response and eventually lead to the destruction of the attachment apparatus and bone loss around a tooth or a dental implant. Such results have a negative impact on an individual's oral health-related quality of life [1, 2].

*Aggregatibacter actinomycetemcomitans* is a facultative anaerobic Gram-negative bacterium that expresses various virulence factors that trigger inflammation in the periodontal tissue [3]. Furthermore, *A. actinomycetemcomitans* is one of the main causative factors of periodontal disease in juveniles and adolescents [4]. In addition to periodontal disease, *A. actinomycetemcomitans* was found to have strong associations with peri-implantitis [5–7].

The goal of treating periodontitis and peri-implantitis is similar: eliminating the bacterial load of periodontal pockets to restore the biological compatibility of periodontally diseased root surfaces and to allow for implant re-osseointegration. However, their treatment is challenging, as routine mechanical debridement does not eliminate completely the load of bacterial strains [8–10]. Use of adjunct antibiotics may increase bacterial clearance. However, the overuse of antibiotics is the main reason for the emergence of drug-resistant bacteria [4]. Therefore, the development of new antimicrobial approaches against periodontal pathogenic bacteria with fewer complications is necessary.

Resolvin E1 (RvE1) is part of the specialized pro-resolving lipid mediator (SPM) family biosynthesized from omega-3, polyunsaturated fatty acids (PUFAs), and eicosapentaenoic acid (EPA). It plays a role in regulating the coordinated termination of inflammation by halting neutrophil infiltration, enhancing the recruitment of resolution monocytes, and providing a negative feedback loop for resolving the acute phase of inflammatory response. SPMs also aid in actively promoting tissue repair, bacterial clearance, and bone remodeling [11, 12]. Although RvE1 is an established anti-inflammatory agent, it was found that its application as a treatment or as a preventive drug had an indirect effect on subgingival microbiota of both rats and rabbits with experimental periodontitis. In prevention experiments, RvE1 helped reduce the shift of subgingival microbial from Gram-positive to Gram-negative bacteria [13]. Such results were intriguing to evaluate RvE1's direct anti-microbial potential. Consequently, the aim of this study is to evaluate the direct antimicrobial effect of RvE1 on *A. actinomycetemcomitans* bacteria.

## Materials and Methods

### Targeted Bacteria and Culture Conditions

*A. actinomycetemcomitans* serotype B was acquired commercially from ATCC^®^ 29522™ (American Type Culture Collection, Manassas, VA, United States) and cultured according to manufacturer's instructions in chocolate agar media with 5–7% CO_2_ for 24–48 h. A single colony was sub-cultured in brain-heart infusion (BHI) (Sigma-Aldrich, Inc. 14508 St. Louis, United States) broth for 24 h. After broth incubation, the concentration was adjusted to 0.5 optical density (OD_600_) with a spectrophotometer (Terra Universal, CA, United States).

For growth curve analysis, 200 ul of the bacterial suspension (0.5 OD_600_) was transferred (in triplicates) to a Honeycomb 100-well plate (Bioscreen C, United States). Plate reading was carried out with a Bioscreen C essay reader set at 35°C for 48 h (Bioscreen C Automation for Microbiology, 11 Blueberry Court, Piscataway, NJ 08854, United States).

### RvE1 Preparation

RvE1 was acquired commercially from Cayman Chemical^®^ (1180 East Ellsworth Road Ann Arbor, Michigan 48108 USA) in a bottle containing 50 μg dissolved in 1 ml of 100% ethanol and stored in −80°C according to manufacturer's instructions. RvE1 was diluted in phosphate-buffered saline (PBS) (Sigma-Aldrich, Inc. 14508 St. Louis, United States) 10 times to achieve a concentration of 5 ug/ml in 10% ethanol and was considered to be the working concentration.

### Treatment Groups

The study comprised three groups that underwent minimum inhibitory concentration (MIC) against *A. actinomycetemcomitans*. The first group was tested with the RvE1 working concentration, the second group was tested with ethanol (EtOH), 10% as the working concentration, and the final group was diluted in PBS as the positive control. Optical density (OD_600_) was used for the comparison of bacterial growth between tested groups. The experiment was conducted in three biological replicates.

### Minimum Inhibitory Concentration

**Figure 1:**
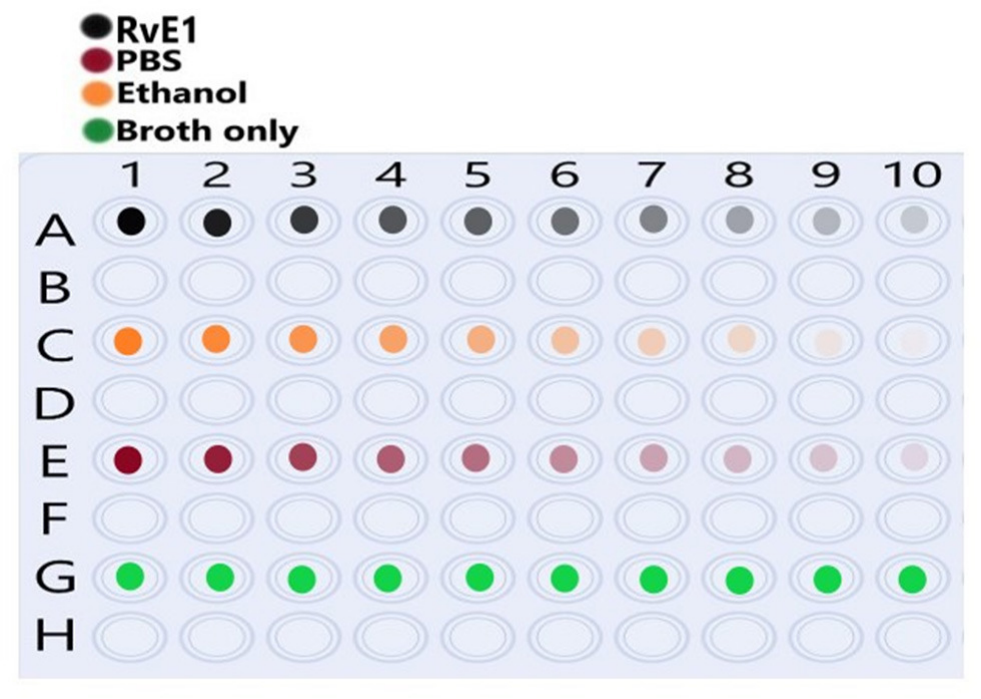
Illustration of the well platesused for serial dilution of each tested group. Row (A) shows Resolvin E1 (RvE1) serial dilution (1:1) from wells 1–10 as color intensity. Row (C) shows ethanol serial dilution (1:1) from wells 1–10 as color intensity. (E) Shows phosphate-buffered saline (PBS) serial dilution (1:1) from wells 1–10 as color intensity. Row (G) shows the brain-heart infusion (BHI) broth medium only without dilution.

The MIC testing was conducted using the broth microdilution method (Figure 1) and according to the standards of the Clinical and Laboratory Standards Institute (CLSI, 2018) [14]. Using 100-well plates, 200 ul of sterile BHI broth was placed in rows A, C, and E (well numbers 2– 9). In well no. A1, 400 ul of the working concentration of Rve1 (5 μg/ml) was added. 200 ul of the working concentration was transferred to wells no. A2 and further serial dilutions (1:1 ratio) of 200 ul up to well no. A10. After dilution of the last well, 200 ul of the solution was discarded. The same protocol was carried out on ethanol (row C) with a working concentration of 10% and the PBS group (row E).

Next, 10 ul of 0.5 OD *A. actinomycetemcomitans* was added in all the wells. In row G, 200 ul of BHI broth in triplicates were added as a negative control. After preparations of wells, the 100-well was inserted into the Bioscreen C essay reader (Bioscreen Automation for Microbiology, 11 Blueberry Court, Piscataway, NJ 08854) set at 35°C for 48 h with hourly reading intervals of the optical density.

### Statistical Analysis

The power of study (85%) was calculated using the G^*^power 3.1.9.7 application software (Heinrich-Heine-University Düsseldorf 40204 Düsseldorf) Using a minimum level of significance (α) of 0.05 with an effect size 05, data were analyzed using SPSS, version 20 (IBM, Somers, NY, United States). All the results were analyzed by one-way analysis of variance (ANOVA) followed by *post-hoc* Bonferroni using a minimum level of significance (*P*-value) of 0.05.

## Results

**Figure 2:**
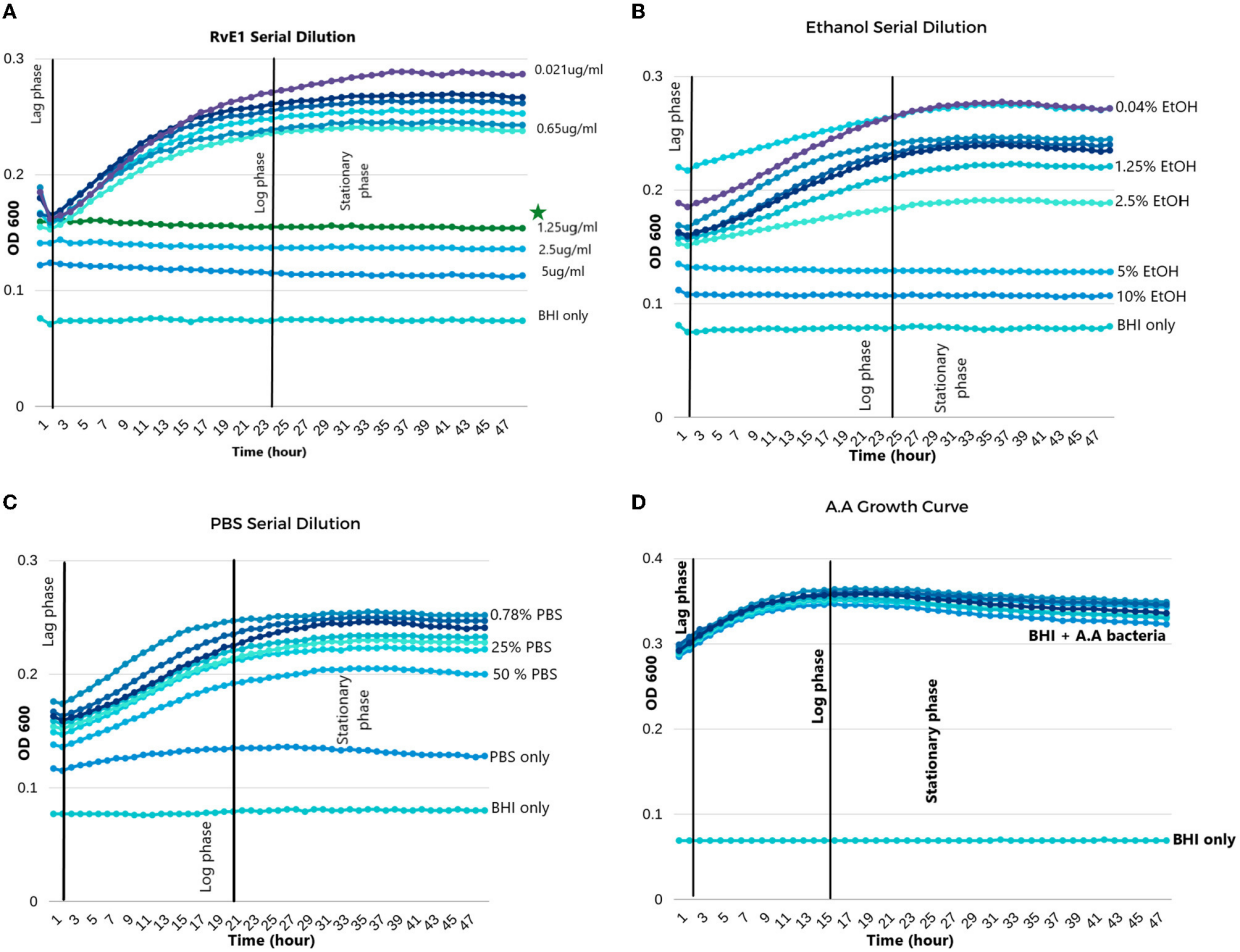
*Aggregatibacter actinomycetemcomitans* growth in different solutions. **(A)** Serial dilution of Resolvin E1 diluted in BHI broth; minimum inhibitory concentration (MIC) was 1.25 μg/ml. **(B)** Serial dilution of ethanol diluted in BHI broth; MIC was 5%. **(C)** Serial dilution of PBS diluted in BHI broth showed growth in all dilutions except for the PBS without BHI broth. **(D)** Growth curve of *A. actinomycetemcomitans* in BHI broth without any dilutions. *The symbol indicates the MIC of RvE1.

*The A.actinomycetemcomitans* cultured in BHI had a growth pattern that entered the lag-phase in the first 2 h, which was followed by the log-phase that reached its climax of growth between 20 and 24 h. In the following 24 h, the bacteria did not grow any further, and it was considered as the stationary phase. During each MIC experiment, the broth-only wells did not encounter any growth, which indicates the lack of cross-contamination between all the wells (Figure 2D).

In the RvE1 group, wells from 1 to 4 did not reveal any bacterial growth patterns and were stagnant during the experiment. Therefore, the MIC of RvE1 was in well no 0.4 with a concentration of 1.25 μg/ml as shown in Figure 2A. On the other hand, the ethanol group (Figure 2B) revealed that concentrations of (10 and 5%) did not enter the log phase with an MIC of 5%, the 2.5% and the following diluted concentrations exhibited similar growth manner to the PBS group (Figure 2C) in which they all entered the normal phases of bacterial growth (lag, log, and stationary).

RvE1's mean OD was (0.156 ± 0.021) and was significantly lower than that of all the other groups (*P*-value < 0.01). The ethanol group (mean OD 0.178 ± 0.013) and the PBS group (mean OD 0.1855 ± 0.022) did not reveal a significant difference (*P*-value = 0.185). Further comparisons between group means are illustrated in Table 1.

**Table 1:** Comparisons of group mean optical density (OD_600_) ± standard deviation.

**Group**	**Against group**	**Mean (OD_**600**_) ± SD**	***P*-value**
RvE1	AA Growth	0.3378 ± 0.016	<0.001
	PBS	0.1855 ± 0.022	<0.001
	EtOH	0.1788 ± 0.013	<0.001
EtOH	AA Growth	0.3378 ± 0.016	<0.001
	PBS	0.1855 ± 0.022	0.185
	RVE1	0.1563 ± 0.021	<0.001

*All the groups showed significant bacterial growth against Resolvin E1 (RvE1), while there was no significant difference between the ethanol group vs. PBS) group*.

During different time intervals (6, 24, and 48 h), we compared the MIC value of RvE1 (1.25 μg/ml), ethanol (2.5%), and PBS against each other and against the *A. actinomycetemcomitans* growth curve. Comparisons are also conducted within each group and are further detailed in Table 2. RvE1 (1.25 μg/ml) 6, 24, and 48 h did not show any difference between them (*P*-value = 1). Additionally, the RvE1 group (1.25 μg/ml) had significantly lower bacterial growth in comparison to *A. actinomycetemcomitans* growth curve, ethanol 2.5%, and PBS during all three time intervals (*P*-value < 0.001). Notably, ethanol 2.5% showed insignificant results along with the PBS group during the 6-, 24-, and 48-h intervals (*P*-value = 1).

**Table 2:** Comparisons of specific concentrations at different time points (6, 24, and 48 h).

**Group**	**Against group**	**Mean (OD) ± SD**	***P*-value**
RvE1 (1.25μg/ml) 6 h	RvE1 (1.25 μg/ml) 24 h	0.163 ± 0.006	1.00
	RvE1 (1.25) 48 h	0.15 ± 0.0	1.00
	ETOH (2.5%) 6 h	0.153 ± 0.005	1.00
	PBS 6 h	0.147 ± 0.006	0.325
ETOH (2.5%) 24 h	ETOH (2.5%) 6 h	0.193 ± 0.05	<0.001
	ETOH (2.5%) 48 h	0.1933 ± 0.05	1.00
	RvE1 (1.25) 24 h	0.163 ± 0.006	<0.001
	PBS 24 h	0.203 ± 0.005	1.00
PBS 48 h	PBS 6 h	0.147 ± 0.006	<0.001
	PBS 24 h	0.203 ± 0.005	<0.001
	RvE1 (1.25 μg/ml) 48 h	0.15 ± 0.0	<0.001
	ETOH (2.5%) 48 h	0.1933 ± 0.05	1.00

*RvE1 (1.25 μg/ml) showed significant differences in all the time periods except for the 6-h time point, against all the tested groups*.

## Discussion

MIC is considered the “gold standard” to determine the antimicrobial ability of a drug against a specific microorganism. MIC is defined as the lowest concentration of an antimicrobial agent that hinders the growth of a particular microorganism under controlled conditions [15, 16].

The results of this study supported our aim in that RvE1 had an antimicrobial effect against *A. actinomycetemcomitans*. The MIC of RvE1 was 1.25 μg/ml. As RvE1 is dissolved in ethanol, ethanol was chosen as a comparative group. RvE1's concentration of 1.25 μg/ml contains 2.5% of ethanol. The statistical significance of the growth of bacteria in the 2.5% ethanol compared to the ceased growth in the 1.25 μg/ml of RvE1 excludes ethanol as a major component for the antimicrobial effect.

In 2021, Elashiry et al. conducted a comprehensive review study on selective antimicrobial therapies for treating periodontitis and known antibiotics, such as metronidazole and amoxicillin, were classified as direct antimicrobial agents. Interestingly, RvE1 was classified as an indirect antimicrobial therapy for periodontitis because of its established anti-inflammatory actions. However, they concluded that further studies are needed to be conducted on RvE1 [17].

To the best of our knowledge, this is the first study demonstrating a significant direct antimicrobial action of RvE1 against *A. actinomycetemcomitans* bacteria. These findings may emerge from RvE1's major components such as polyunsaturated fatty acids (PUFAs) and eicosapentaenoic acid (EPA). Although they are established as anti-inflammatory compounds, their antimicrobial potential has become an area of interest in recent years. Huang et al. conducted a novel study evaluating the antimicrobial effect of various acids derived from PUFAs against oral pathogens. They found that PUFA derivatives had a remarkable inhibitory action against oral pathogens such as *A. actinomycetemcomitans, Fusobacterium nucleatum*, and *Porphyromonas gingivalis*. The exact mechanism of the fatty acids' antimicrobial action is unclear. However, Haung et al. described that the lipid membrane of fatty acids has a hydrophilic head and a hydrophobic tail that is similar to that of a bacterial cell wall membrane. Therefore, fatty acids may penetrate bacteria by targeting the cell membrane and disrupting it [18]. In 2016, Sun et al. evaluated the antimicrobial effect of eicosapentaenoic acid (EPA) against *P. gingivalis* bacteria. They found that EPA demonstrated a substantial inhibitory effect against *P.gingivalis* with an MIC of 12.5 μM; an EPA concentration of 100 μM completely killed *P.gingivalis*, and when the species of bacteria was observed under a scanning electron microscope, they found that its cell membrane was completely disrupted, which led to bacterial lysis [19].

Although our results reflect a direct antimicrobial effect of RvE1 in a controlled *in vitro* environment, it may not express the same effect in a clinical situation where host response and other local/systemic factors play a role in complex inflammatory conditions such as periodontitis and peri-implantitis. Therefore, further preclinical and clinical studies are needed to establish RvE1 as an adjunctive treatment modality for periopathogenic bacteria.

## Conclusion

In conclusion, RvE1 demonstrated significant antimicrobial activity against *A. actinomycetemcomitans* at an MIC of 1.25 μg/ml. The RvE1 group showed significantly lower bacterial growth compared to the EtOH and PBS groups.

## Data Availability Statement

The raw data supporting the conclusions of this article will be made available by the authors, without undue reservation.

## Author Contributions

FA: conceptualization and writing—original draft preparation. FA, MA-A, and BA: methodology. MA-A and BA: validation. MA: formal analysis, writing—review and editing, and supervision. FA and BA: data collection. All authors have read and agreed to the published version of the manuscript.

## Conflict of Interest

The authors declare that the research was conducted in the absence of any commercial or financial relationships that could be construed as a potential conflict of interest.

## Publisher's Note

All claims expressed in this article are solely those of the authors and do not necessarily represent those of their affiliated organizations, or those of the publisher, the editors and the reviewers. Any product that may be evaluated in this article, or claim that may be made by its manufacturer, is not guaranteed or endorsed by the publisher.
